# Elucidating the molecular determinants in the process of gastrin C-terminal pentapeptide amide end activating cholecystokinin 2 receptor by Gaussian accelerated molecular dynamics simulations

**DOI:** 10.3389/fphar.2022.1054575

**Published:** 2023-01-23

**Authors:** Kecheng Yang, Huiyuan Jin, Xu Gao, Gang-Cheng Wang, Guo-Qiang Zhang

**Affiliations:** ^1^ National Supercomputing Center in Zhengzhou, Zhengzhou University, Zhengzhou, China; ^2^ School of International Studies, Zhengzhou University, Zhengzhou, China; ^3^ Department of General Surgery, Affiliated Cancer Hospitalof Zhengzhou University, Henan Cancer Hospital, Zhengzhou, China

**Keywords:** gastrin, cholecystokinin 2 receptor, interaction mechanism, GAMD, PMF, MM-PBSA analysis

## Abstract

Gastrin plays important role in stimulating the initiation and development of many gastrointestinal diseases through interacting with the cholecystokinin 2 receptor (CCK2R). The smallest bioactive unit of gastrin activating CCK2R is the C-terminal tetrapeptide capped with an indispensable amide end. Understanding the mechanism of this smallest bioactive unit interacting with CCK2R on a molecular basis could provide significant insights for designing CCK2R antagonists, which can be used to treat gastrin-related diseases. To this end, we performed extensive Gaussian accelerated molecular dynamics simulations to investigate the interaction between gastrin C-terminal pentapeptide capped with/without amide end and CCK2R. The amide cap influences the binding modes of the pentapeptide with CCK2R by weakening the electrostatic attractions between the C-terminus of the pentapeptide and basic residues near the extracellular domain in CCK2R. The C-terminus with the amide cap penetrates into the transmembrane domain of CCK2R while floating at the extracellular domain without the amide cap. Different binding modes induced different conformational dynamics of CCK2R. Residue pairs in CCK2R had stronger correlated motions when binding with the amidated pentapeptide. Key residues and interactions important for CCK2R binding with the amidated pentagastrin were also identified. Our results provide molecular insights into the determinants of the bioactive unit of gastrin activating CCK2R, which would be of great help for the design of CCK2R antagonists.

## 1 Introduction

Gastrin is a peptide hormone that can regulate gastric acid secretion and stimulate gastric mucosal growth (Dockray, 2004; Dimaline and Varro, 2014). Many variants of gastrin with various amino acid lengths are existing in the human body and the main forms contain 17 or 34 amino acid residues with an amidated C-terminus (amidated gastrins) (Calatayud et al., 2010; Dimaline and Varro, 2014). Amidated gastrins with different amino acid lengths have full biological activities but varied circulating half-lives, while non-amidated forms have almost no biological activities (Calatayud et al., 2010; Dimaline and Varro, 2014). The essential bioactive unit of gastrin is confirmed as the C-terminal tetrapeptide amide sequence (Trp-Met-Asp-Phe-NH_2_) (Dockray, 2004). Gastrin exerts its biological functions *via* binding to cholecystokinin 2 receptor (CCK2R), a member of class A G protein-coupled receptor (GPCR) superfamily. CCK2R has another endogenous ligand, CCK, which shares the same C-terminal five residues and amide end as gastrin and is responsible for pancreatic enzyme secretion (Calatayud et al., 2010; Dimaline and Varro, 2014). CCK and gastrin are the only family members of regulatory peptides in mammals (Rehfeld, 2017). The bioactive unit of CCK is its C-terminal seven amino acids amide sequence (Rehfeld, 2017). Apart from the normal physiology effects of gastrin activating the CCK2R signaling pathway, it is also found that CCK2R can be activated by gastrin in many gastrointestinal diseases including peptic ulcer disease, malignant tumors of the colon, pancreatic and gastric cancers, resulting in cell proliferation (Fourmy et al., 2011; Dimaline and Varro, 2014; Maddalo et al., 2014; Smith et al., 2016). Among these diseases, gastric and pancreatic cancers are the third and seventh leading causes of cancer deaths, respectively (Bray et al., 2018). Interrupting the interaction between gastrin and CCK2R should be feasible to treat these deadly cancers. However, despite many strategies that have been explored to develop CCK2R antagonists, there are still no approved drugs. At present, surgical resection is the only effective therapy to treat these malignancies (Gilliam and Watson, 2007; Hussain, 2016; Bray et al., 2018; Dolcetti et al., 2018; Sitarz et al., 2018). Understanding the mechanism of gastrin interacting with CCK2R as well as the dominant role of the C-terminal amide cap should give significant insights into developing CCK2R antagonists.

Considerable experiments have been performed to explore the activation mechanism of CCK2R in the past 30 decades (Silvente-Poirot and Wank, 1996; Bläker et al., 1998; Silvente-Poirot et al., 1999; Pannequin et al., 2002; Irina et al., 2007; Stone et al., 2007; Foucaud et al., 2008; Song et al., 2013; Ritler et al., 2019). Methods include modifying residues in CCK2R, gastrin and CCK to identify the key residues accounting for interaction. It is found that gastrin with sulfated or non-sulfated tyrosine has similar binding affinities with CCK2R (Dockray, 2004; Dimaline and Varro, 2014). The negatively charged pentaglutamic acid sequence in the gastrin is important for gastrin’s stability in human blood plasma and can increase its binding affinity with CCK2R. Segment replacement combined with site-directed mutagenesis identified five residues (Q^204^CVHRW^209^) in the second extracellular loop of rat CCK2R playing major roles in the selectivity of gastrin (Silvente-Poirot and Wank, 1996). Point mutations also found some residues in CCK2R can significantly alter the activity of gastrin and CCK(Silvente-Poirot et al., 1998; Anders et al., 1999; Bläker et al., 2000; Galés et al., 2003; Irina et al., 2007; Foucaud et al., 2008; Willard et al., 2012; Magnan et al., 2013; Lipiński et al., 2018). Very recently, three cryo-EM structures of CCK2R in complex with gastrin-17 and G_q_ or G_i2_ (PDB codes: 7F8W, 7XOW and 7F8V) were obtained, which presented the complex conformations at atomic level (Zhang et al., 2021; Ding et al., 2022). Besides experiment methods performed to identify key sites contributing to gastrin/CCK-CCK2R interactions, computation methods including docking and molecular dynamics (MD) simulations were also performed to delineate the complete binding pose and analyze the main interactions (Langer et al., 2005; Marco et al., 2007; Stone et al., 2007; Foucaud et al., 2008; Magnan et al., 2013). However, these MD results are limited by the simulation time of tens of ns which may not be sufficient for conformational adjustment from the initial docking pose. Besides, the molecular dissection of the indispensable role of the amide cap still remains elusive.

Benefiting from the encouraging development of computational power and computational methods (Miao and McCammon, 2016; Mori et al., 2016; Yang et al., 2016; Eastman et al., 2017; Miao and McCammon, 2017; Yang et al., 2019), the whole processes of protein folding, biomolecular large-scale conformational transitions and biomolecular recognition in the explicit solvent can be simulated within an acceptable time (Lindorff-Larsen et al., 2011; Eastman et al., 2017; Miao and McCammon, 2017; Palermo et al., 2017). In the present work, we applied Gaussian accelerated MD (GaMD) simulations (Pang et al., 2017; Bhattarai et al., 2020), an advanced conformational sampling method, to explore the influence of the amide cap on the conformational dynamics of gastrin C-terminal pentapeptide as well as its binding modes with CCK2R. We showed the molecular basis of the gastrin bioactive unit interacting with CCK2R and the mechanism of the indispensable role of the amide cap in gastrin fulfilling its physiological functions. These results should provide deep understanding of the activation mechanism of CCK2R and practical guides for designing CCK2R antagonists.

## 2 Materials and methods

### 2.1 System preparation

CHARMM-GUI (Jo et al., 2008) was used to prepare the simulation systems from the cryo-EM structure of CCK2R in complex with gastrin-17 (PDB code: 7F8W) (Zhang et al., 2021). First, the bioactive unit structure of C-terminal pentagastrin (Gly-Trp-Met-Asp-Phe) was truncated from the structure of gastrin-17 and the amidated (termed as G5NH_2_) and non-amidated (termed as G5) C-termini were built. The neutral N-terminus was used for G5/G5NH_2_ to imitate the primitive form of pentagastrin in gastrin-17 and minimize the influence of the N-terminus on the binding modes. Besides, the disulfide bond between Cys127^3.25^ and Cys205^ECL2^ (superscript indicates nomenclature according to Ballesteros—Weinstein numbering system (Isberg et al., 2015)) in CCK2R was also built. Then the Membrane Builder plugin (Wu et al., 2014) was used to pack the explicit POPC lipid bilayer around CCK2R-G5 and CCK2R-G5NH_2_ complexes, respectively. Subsequently, 20 Å thickness TIP3P water molecules were placed above and below the lipid bilayer. The protonation states of all residues in G5, G5NH_2_ and CCK2R were set at PH 7. Additional Na^+^ and Cl^−^ ions were added to all simulation systems to keep the environment neutral and the salt concentration at 0.15 M.

### 2.2 Simulation protocols

All simulations were performed by NAMD v2.13 (Phillips et al., 2005) with GPU acceleration. Protein and lipid atoms were parameterized with CHARMM36m force field (Huang et al., 2017). Periodic boundary conditions were applied in all three directions. Short-range non-bonded interactions were computed every step using a cutoff of 12 Å with a switch distance of 10 Å. Long-range electrostatic interactions were calculated every two steps using the Particle-Mesh Ewald (PME) (Essmann et al., 1995) method with a grid spacing of 1 Å^−1^. The integration time step was set to 2 fs with all bonds involving hydrogen atoms constrained by the SHAKE algorithm (Weinbach and Elber, 2005). Constant temperature of 300 K and constant pressure of 1 atm were kept by langevin dynamics and Nosé-Hoover Langevin piston method (Feller et al., 1995), respectively.

All systems were firstly undergone 5,000 steps of conjugate gradient energy minimization to remove the steric clashes in the system. Then 300 ps gradually heating process to rise the temperature from 0 to 300 K in the NVT-ensemble and 1 ns NPT-ensemble MD simulations used to adjust the volume of each system were performed. In the last two steps, harmonic potential with the force constant of 1.0 kcal/mol/Å^2^ was applied to all non-hydrogen atoms of solutes to minimize their structures change. Another 1 ns NPT-ensemble MD simulations were performed with the harmonic potential gradually decreased to 0 kcal/mol. Finally, GaMD simulations were carried out for thoroughly conformational sampling.

GaMD is an unconstrained enhanced conformational sampling technique which works by adding a harmonic boost potential to reduce the energy barriers at the system’s potential surface. When the system potential energy (V) is lower than the threshold energy E, a boost potential (ΔV) is added and the system potential is modified as.

$$V^{*}=V+\Delta V$$

$$\Delta V=\left\{\begin{array}{cc} \frac{1}{2}k{\left(E-V\right)}^{2}, & V<E,\\ 0, & V\geq E, \end{array}\right.$$ (1)

where k is the harmonic force constant which determines the strength of ΔV . And the strength of ΔV should satisfy the criterion that the modified potential energy surface does not change the relative order of potential values in the original potential energy surface (i.e., if V1 < V2, then V1* < V2*). In the present work, the threshold energy E was set equal to the system’s maximum potential energy. The maximum potential energy and the potential energy surface of the system were estimated in a 2 ns conventional MD simulation protocol, at the same time, the value of k was determined automatically based on E and the system’s potential energy surface. Then dual-boost GaMD simulations with default parameters were performed, which included 50 ns equilibration runs and two independent 1 μs production runs for all systems. The two independent production simulations were started with different randomized initial atomic velocities. Trajectory frames were saved every 4 ps for analysis.

### 2.3 Trajectory analysis

Based on the GaMD production simulations, the 2D potential of mean force (PMF) profiles were calculated using PyReweighting toolkit (Miao et al., 2014) with the reweighting method of cumulant expansion to the second order. The two parallel GaMD trajectories of each system were combined first before reweighting. Two reaction coordinates were defined as distances between Cα atoms in N/C-terminal residues in G5/G5NH_2_ and the geometrical center of the transmembrane domain (calculated for backbone atoms) of CCK2R. The residues ID of each transmembrane domain in CCK2R is listed in Supplementary Table S1. Principal component analysis (PCA) was performed using the Wordom software (Seeber et al., 2011) to differentiate the dominant conformational features of CCK2R induced by G5 and G5NH_2_ binding. The correlations of the atomic fluctuations in the CCK2R-G5/G5NH_2_ complex are calculated by the Linear Mutual Information (LMI) (Lange and Grubmüller, 2006) method within the Bio3D package (Grant et al., 2021).

Residues contributing to CCK2R-G5/G5NH_2_ binding were denoted by MM-PBSA method with MMPBSA.py program (Miller et al., 2012):

$$\begin{array}{l} \Delta G_{binding}=\Delta G_{complex}-\left(\Delta G_{receptor}+\Delta G_{ligand}\right)\\ \Delta G=\Delta E_{gas-MM}+\Delta G_{solvation-PBSA}-T\Delta S\\ =\left(\Delta E_{gas-vdw}+\Delta E_{gas-ele}\right)+\left(\Delta G_{sol-polar}+\Delta G_{sol-nonpolar}\right)-T\Delta S \end{array}$$ (2)

where $\Delta E_{gas-vdw}$ and $\Delta E_{gas-ele}$ are the van der Waals and electrostatic components of molecular mechanical energies (MM) in the gas phase, ${\Delta G}_{sol-polar}$ and ${\Delta E}_{sol-nonpolar}$ are the polar and non-polar contributions of solvation free energies calculated using the implicit solvent model of PBSA. ΔS is the entropic contribution which was ignored in the present work due to the difficulty in computation, low reliability (Cui et al., 2015; Wang et al., 2018; Zhang et al., 2018), and our main target is to distinguish the key residues for binding. The dielectric constants of membrane, solute and solvent were set to 1.0, 6.0 and 80.0, respectively (Hou et al., 2011). The total binding free energy was decomposed into the per-residue level to quantitatively evaluate the contribution of each residue.

The binding sites are defined as the atoms of CCK2R located within 4.0 Å of the heavy atoms in G5/G5NH_2_. In the MD trajectory frames, the occurrence rates of each heavy atom located in the binding sites were calculated, and one residue is denoted as in the binding sites only if the sum of the occurrence rates of every heavy atom in it is larger than 1. Key interactions of hydrogen bonds and salt bridges (Yang et al., 2018) formed between CCK2R and G5/G5NH_2_ are also analyzed. The hydrogen bond is detected by VMD according to the criteria of the distance of donor-acceptor less than 3.5 Å and the angle of donor-H-acceptor larger than 150°. The salt bridge is defined as the distance between the oxygen atom of the carboxyl group and the nitrogen atom at the side chain of basic residues (Arg and Lys) is less than 4.0 Å. Besides, the occurrence rates of hydrogen bonds and salt bridges in the trajectory frames should be larger than 0.5. Residues which locate in the binding site and made binding free energy contributions larger than 1 kcal/mol are denoted as key residues for binding.

## 3 Results and discussion

### 3.1 Free energy profiles of CCK2R-G5 and CCK2R-G5NH_2_

**FIGURE 1:**
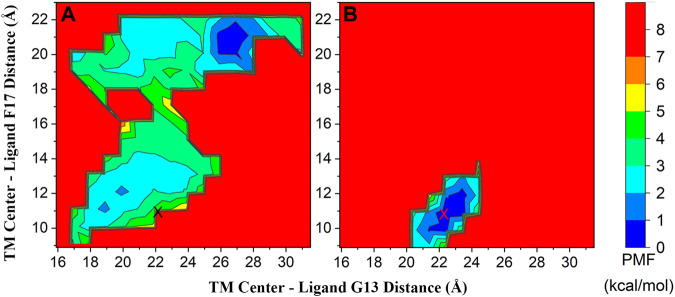
2D PMF profiles of CCK2R-G5 **(A)** and CCK2R-G5NH_2_ **(B)** along the reaction coordinates of distances between the N/C-terminus of G5/G5NH_2_ (Cα atoms of G13 and F17, respectively) and the geometrical center of TM domain of CCK2R; The initial site from the cryo-EM structure was marked as symbol X (coordinate: 22.1, 10.8). The PMF profile of each system was calculated by reweighting the combined two 1 μs GaMD simulations.

To clarify the binding mechanism of the smallest bioactive unit of gastrin to CCK2R and why the C-terminal amide cap is indispensable for gastrin activating CCK2R, we performed a total of 2 μs (two individual 1 μs trajectories) GaMD production simulations on the CCK2R-G5 and CCK2R-G5NH_2_ complexes, respectively. From the GaMD production simulations, 2D PMF profiles along the reaction coordinates of distances between the N/C-terminus of G5/G5NH_2_ (Cα atoms of G13 and F17, respectively) and the geometrical center of the TM domain of CCK2R were calculated and presented in Supplementary Figures S1, S2 and Figure 1. Supplementary Figure S1 shows that the lowest free energy regions with PMF less than 1 kcal/mol are very similar from 800 ns to 1 μs (combined two parallel trajectories), which suggests the convergence of present GaMD simulations. Besides, the lowest PMF regions for CCK2R-G5NH_2_ complexes are around the initial site in the cryo-EM conformation, while having some distance from the initial site for CCK2R-G5 complexes (Figures 1A, B). Supplementary Figure S2 shows the PMF profiles calculated from the individual trajectory. From Supplementary Figures S2A, B, we can find that different minimal PMF regions were obtained from different trajectories for the CCK2R-G5 system. To find why the PMF files calculated from individual trajectories differed, we depicted the time evolutions of two reaction coordinates in each trajectory in Supplementary Figure S3. From Supplementary Figure S3A, we can find that for the reaction coordinate of TM center—G13 distance, a significant difference occurred after 150 ns between two trajectories, which indicates different conformational spaces sampled by two trajectories. However, both reaction coordinates have stable fluctuations after 400 ns (Supplementary Figures S3A, B). PMF profiles calculated from merged trajectories (Figure 1A) showed that the minimal PMF region obtained in trajectory 1 (Supplementary Figure S2A) was lower than that obtained in trajectory 2 (Supplementary Figure S2B). To further verify the minimal PMF region found by trajectory 1, we performed another 300 ns simulations (termed trajectory 3) from the conformational state at 700 ns of trajectory 1 with different randomized initial atomic velocities. The calculated PMF profile is depicted in Supplementary Figure S2C, and the time evolutions of two reaction coordinates are depicted in Supplementary Figure S3. We can find that the minimal PMF region and the time evolutions of two reaction coordinates in trajectory 3 are much similar to those in trajectory 1. Adding trajectory 3 hardly affects the calculated PMF profiles (Supplementary Figure S2D), which indicates that the two parallel 1 μs simulations are adequate for the CCK2R-G5 systems. For the CCK2R-G5NH_2_ system, the PMF profiles and the time evolutions of two reaction coordinates calculated from each trajectory are much similar (Supplementary Figures S2, S3). Both reaction coordinates for two trajectories have stable fluctuations in the whole 1 μs simulation time, which indicates the initial conformational state was very steady. However, to further verify the minimal PMF region for the CCK2R-G5NH_2_ system, we performed another two parallel 700 ns simulations (termed trajectory 3 and 4) from the same initial conformational state with different randomized initial atomic velocities. We can find that the minimal PMF region and the time evolutions of two reaction coordinates are much similar for four parallel trajectories (Supplementary Figure S2E–H). Moreover, adding trajectories 3 and 4 also hardly affects the calculated PMF profiles (Supplementary Figure S2I), which indicates that the two parallel 1 μs simulations are also adequate for the CCK2R-G5NH_2_ systems.

To see how the complex structures changed along with the simulation time, we depicted the time evolutions of RMSD values of simulation structures of CCK2R (based on TM backbone atoms) and G5/G5NH_2_ (based on backbone atoms) from their initial structures (aligned with TM backbone atoms, Supplementary Figure S4). We can find that structures of CCK2R and G5NH_2_ were relatively stable in the whole 1 μs simulations in each trajectory, while structures of G5 endured larger changes, especially for those in CCK2R-G5 trajectory 1. Those results also indicated that the initial conformational state for CCK2R-G5NH_2_ was in an energy-stable state but for CCK2R-G5 was in an energy-unstable state.

To make a detailed comparison between the binding modes and the underlying mechanisms of CCK2R in complexes with G5 and G5NH_2_, we extracted about 5,000 frames with PMF values equal to 0 kcal/mol evenly from the two parallel 1 μs simulations for the following analysis.

### 3.2 Conformational dynamics of CCK2R in complex with G5/G5NH_2_

**FIGURE 2:**
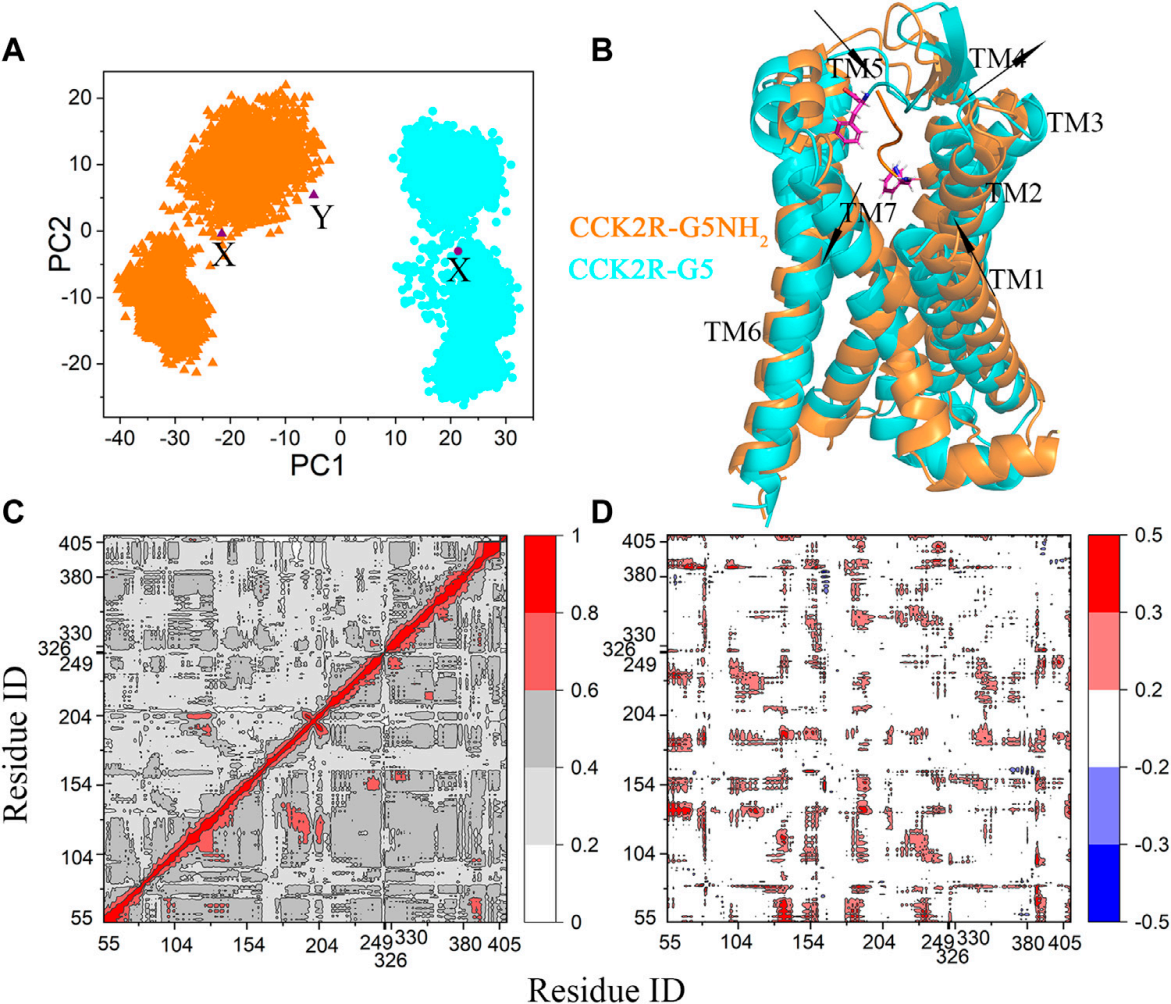
**(A)** Projections of CCK2R conformations in the complexes of CCK2R-G5 (cyan dots) and CCK2R-G5NH_2_ (orange triangles) with PMF equal to 0 kcal/mol onto the common subspace defined by the first two PCs. The representative conformations in B are marked as symbol X, and the cryo-EM structure is marked as symbol Y. **(B)** Comparison of representative conformations of CCK2R-G5 (cyan) and CCK2R-G5NH_2_ (orange) complexes with PMF equal to 0 kcal/mol. CCK2R is shown as cartoons. G5 and G5NH_2_ are shown as cartoons, and their C-termini are shown as sticks. The directions of TM movement of CCK2R along with the PC1 from lowest to highest are indicated by arrows. **(C)** Linear mutual information (LMI) correlations between Cα atom pairs within CCK2R-G5 (upper triangle) and CCK2R-G5NH_2_ (lower triangle) complexes. Residues from 55 to 405 belong to CCK2R, and the rest 5 (at the end of the coordinate axis) belong to G5/G5NH_2_. **(D)** Differences in correlation coefficients between CCK2R-G5NH_2_ and CCK2R-G5. The correlation coefficient is color-coded (see color bars).

PCA was performed (based on the Cα atoms of CCK2R) to investigate whether the primary conformational features of CCK2R in complexes with G5 and G5NH_2_ were the same. To make the dynamics of CCK2R conformations can be compared within a common subspace, the CCK2R conformations extracted from the CCK2R-G5 and CCK2R-G5NH_2_ complexes were combined and aligned with the cryo-EM structure based on the Cα atoms of TM domain first. The projections of CCK2R conformations onto the first two PCs were presented in Figure 2A. We can see that an obvious difference occurred at PC1 (the most important principal component of conformational dynamics), with negative and positive values for CCK2R in complexes with G5NH_2_ and G5, respectively. Different PC1 values indicate that the primary conformational features of CCK2R binding with G5 and G5NH_2_ are significantly different. However, CCK2R in cryo-EM structure (binding with gastrin-17) and CCK2R-G5NH_2_ simulation structures were located in the same region (Figure 2A), indicating that CCK2R binding with gastrin-17 and G5NH_2_ has similar primary conformational features. To clearly illustrate the primary structural differences between CCK2R binding with G5 and G5NH_2_, structural motions of CCK2R along with the PC1 were depicted in Supplementary Figure S5A. Form Supplementary Figure S5A, we can find that relative to binding with G5NH_2,_ the upward parts of TM1, 2 and 5 of CCK2R binding with G5 moved closer to the center of the TM bundle. In contrast, other TMs moved away from the center of the TM bundle. Conformations with median PC1 and PC2 values were selected as the representative conformations and were depicted in Figure 2B. We can see that G5NH_2_ stayed in the TM domain of CCK2R while G5 moved out to the extracellular domain. The departure of G5 from the TM domain may be why the upward parts of TM1 and TM2 moved closer to the center of the TM bundle. Besides, conformations of the CCK2R-G5NH_2_ complex obtained in our GaMD simulations with minimal PMF values were much similar to the corresponding parts in the cryo-EM structure (Supplementary Figure S5B). The mean RMSDs of CCK2R (calculated on the TM backbone atoms) and G5NH_2_ (calculated on backbone atoms) from the cryo-EM structure (aligned with the TM backbone atoms) were 2.37 ± 0.39 Å and 1.70 ± 0.35 Å, respectively. At the same time, the minimal RMSD values were 1.51 Å and 1.25 for CCK2R and G5NH_2_, respectively.

Correlated motions of residue pairs in the complexes were calculated by the Linear Mutual Information (LMI) method and depicted in Figure 2C. For clarity, the differences in correlation coefficients between CCK2R-G5NH_2_ and CCK2R-G5 were depicted in Figure 2D. LMI has the merit of no unwanted dependency on the relative orientation of the fluctuations which the Pearson coefficient suffers from (Grant et al., 2021). From Figure 2C, D, we can find that residue pairs have stronger correlated motions in the CCK2R-G5NH_2_ complex than in the CCK2R-G5 complex, which indicates that structural fluctuations induced by G5NH_2_ binding are much easier to transmit to the intracellular parts than by G5 binding.

### 3.3 Binding free energies of G5/G5NH_2_ to CCK2R

The total binding free energies and the contributions of each energy component of G5/G5NH_2_ to CCK2R were calculated by the MM-PBSA method (Miller et al., 2012) and listed in Table 1. By investigating the contributions of each energy component listed in Table 1, we found that the hydrophobic interactions (comprised of ΔG_gas-vdw_ and ΔG_sol-nonpolar_) played dominant roles in the total binding free energies (ΔG_binding_) both for G5 and G5NH_2_. The dominant role of the hydrophobic interactions in the total binding free energy is also found in other protein-protein complexes formation (Simões et al., 2017; Macalino et al., 2018). To further investigate why G5 moved away from the preferred binding sites for G5NH_2_, we performed MM-PBSA analysis on the CCK2R-G5 complexes with similar binding modes as CCK2R-G5NH_2_ complexes. The corresponding results are listed in Supplymentary Table S2. We can find that for the similar binding modes, the hydrophobic interaction for binding with G5 is 12.23 kcal/mol larger than binding with G5NH_2_. This difference was much more significant than the difference in electrostatic interactions (−3.70 kcal/mol, comprised of ΔG_gas-ele_ and ΔG_sol-polar_), which indicates that the TM bundle is unfavorable for ligands binding with many net negative charges.

**TABLE 1:** MM-PBSA derived binding free energies (in unit of kcal/mol) of G5 and G5NH_2_ to CCK2R. MM-PBSA was calculated on the conformations with minimal PMF value.

Complex	ΔG_gas-vdw_	ΔG_gas-ele_	ΔG_sol-nonpolar_	ΔG_sol-polar_	ΔG_binding_
CCK2R-G5	−40.11 (0.30)	−114.70 (0.06)	−6.11 (0.00)	107.79 (0.05)	−53.12 (0.29)
CCK2R-G5NH_2_	−58.35 (0.10)	−61.95 (0.05)	−6.83 (0.00)	60.30 (0.03)	−66.83 (0.09)

The standard error of the mean of the energy is shown in parentheses.

### 3.4 Mechanisms of different binding modes of CCK2R with G5 and G5NH_2_

**FIGURE 3:**
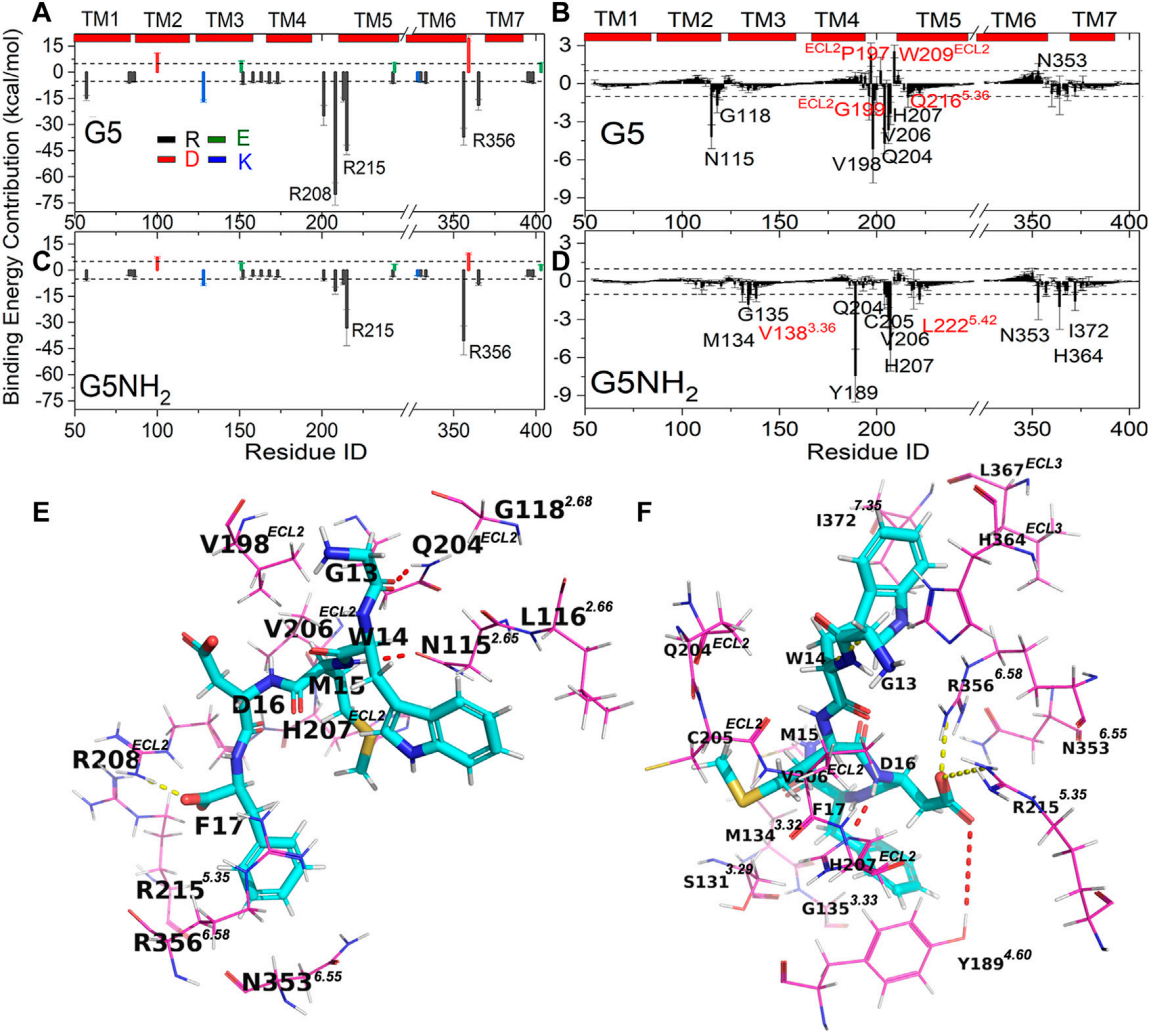
**(A–D)** Free energy (in unit of kcal/mol) contributions of residues in CCK2R for binding with G5 **(A,B)** and G5NH_2_ **(C,D)**. Charged residues (Asp, Glu, Arg and Lys) in CCK2R **(A,C)** located in the binding sites were labeled. Uncharged residues in CCK2R **(B,D)** with absolute values of binding free energy contributions larger than 1 kcal/mol were labeled, in which residues located in and outside binding sites were labeled in black and red, respectively. Dashed lines in A and C, B, and D are the reference lines with values of ±5 and ±1, respectively. Detailed binding poses of CCK2R-G5 **(E)** and CCK2R-G5NH_2_ **(F)**. Residues of G5 and G5NH_2_ are shown in stick representation. Residues of CCK2R are shown in line representation, Salt bridges (R208^ECL2^-F17 in E, R356^6.58^-D16 and R215^5.36^-D16 in F) and hydrogen bonds (N115^2.65^-M15, and Q204^ECL2^-G13 in E, Y189^4.60^-D16 and H207^ECL2^-D16 in F) between CCK2R and G5/G5NH_2_ are depicted by yellow and red dotted lines, respectively.

To shed light on the dominant interactions responsible for the different binding modes of CCK2R with G5 and G5NH_2_, we decomposed the total binding free energy into the per-residue level (Figure 3). Due to the negatively charged pentapeptide of G5 and G5NH_2_, the absolute contributions of all charged residues in CCK2R are larger than 1 kcal/mol and, positively charged residues made favorable contributions, negatively charged residues made unfavorable contributions, respectively. Besides, due to the one more negative charge in G5, charged residues in CCK2R had apparent larger contributions (favorable or unfavorable) for binding with G5 than G5NH_2_ (Figures 3A–C). Another difference is the largest favorable contributions made by charged residues in CCK2R are Arg208^ECL2^ and Arg356^6.58^ for binding with G5 and G5NH_2_, respectively, which also indicated different binding sites for G5 and G5NH_2_. Besides, there are three uncharged resides (Pro197^ECL2^, Trp209^ECL2^, Asn353^6.55^) in CCK2R that made unfavorable contributions larger than 1 kcal/mol for binding with G5 but none for binding with G5NH_2_ (Figures 3B–D), which indicated there existed large unfavorable interactions between CCK2R and G5. By inspecting the per-residue contribution in G5 and G5NH_2_ (Supplymentary Table S3), we found that large differences occurred at the C-terminal two residues of Asp and Phe. The contribution of Asp in G5NH_2_ is more than 20 kcal/mol larger than in G5, while the contribution of the non-amidated Phe in G5 is more than 150 kcal/mol larger than the amidated Phe in G5NH_2_.

By observing the distributions of charged residues in CCK2R and G5/G5NH_2_ complexes, we found that relative to the single charged residue of Asp in G5NH_2_, charged residues of Asp and Phe in G5 were much closer to the positively charged Arg cluster (208^ECL2^, 213^5.33^, 215^5.35^, 356^6.58^, 365^ECL3^) in CCK2R (Supplementary Figure S6). In particular, Arg208^ECL2^ changed to the flat state pointing to G5 from the erect state pointing to solvent. By further investigating the atomic interactions between CCK2R and G5/G5NH_2_ (Figures 3E, F), we found that Arg208^ECL2^ formed a salt bridge with the C-terminal carboxyl of Phe in G5 but no residues formed salt bridges with the sidechain carboxyl of Asp, while Arg356^6.58^ and Arg215^5.35^ formed salt bridges with the sidechain carboxyl of Asp in G5NH_2_. However, Arg356^6.58^ and 215^5.35^ were also located in the binding sites near Phe in G5 (Figure 3E), and the total contributions made by them two were larger for binding with G5 (−81.65 kcal/mol) than binding with G5NH_2_ (−73.35 kcal/mol). The strong electrostatic attractions between the Arg cluster in CCK2R and Phe and Asp in G5 made the C-terminus of G5 move out from the initial binding sites, which were favorable for G5NH_2_. Besides, Gln204^ECL2^ and Asn115^2.65^ formed hydrogen bonds with Gly and Met of G5, respectively, both of which made the top three largest favorable contributions among uncharged residues for CCK2R binding with G5. The time evolutions of distances between atomic pairs involved in the three key interactions between CCK2R and G5 also showed that those three key interactions were very stable in the last 600 ns simulation time in trajectory 1 (Supplementary Figure S7E), which indicated that they played key roles in the stabilize the conformation of G5. However, these binding modes counted against forming salt bridges or hydrogen bonds between Arg208^ECL2^, 215^5.35^, 356^6.58^, Tyr189^4.60^, and His207^ECL2^ (forming hydrogen bonds with Asp in CCK2R-G5NH_2_, see below) and Asp in G5 (Supplementary Figure S7).

G5NH_2_ includes the essential bioactive unit of gastrin activating CCK2R. Thus, the interaction mechanism between G5NH_2_ and CCK2R can help us understand the activation mechanism of CCK2R. Binding conformations with the lowest PMF values in GaMD simulations are much similar to the counterparts in the static cryo-EM conformation (Figure 1B and Supplementary Figure S5B). Arg356^6.58^ and Arg215^5.35^ formed salt bridges with Asp, making the largest two favorable contributions. 12 uncharged residues made favorable contributions with absolute values larger than 1 kcal/mol, 10 of them were located in the binding sites (Figures 3D,F) and another 2 (Val138^3.36^ and Leu222^5.42^) were in the vicinity of binding sites with occurrence rates of 0.7 and 0.2, respectively. Among the uncharged residues, Tyr189^4.60^ and His207^ECL2^ made the first two largest favorable contributions for binding and both of them formed hydrogen bonds with Asp in G5NH_2_. For 14 residues located in the binding sites (Figure 3F), only two made favorable contributions with absolute values less than 1 kcal/mol (-0.94 and -0.74 for Ser131^3.29^ and Leu367^ECL3^, respectively). Thus, 12 key residues (Met134^3.32^, Gly135^3.33^, Tyr189^4.60^, Gln204^ECL2^, Cys205^ECL2^, Val206^ECL2^, His207^ECL2^, Arg215^5.35^, Asn353^6.55^, Arg356^6.58^, His364^ECL3^ and Ile372^7.35^, see methods) for binding with G5NH_2_ are distinguished. Among them, Arg356^6.58^, Arg215^5.35^, Tyr189^4.60^, and His207^ECL2^ played dominant roles in constraining the position of Asp in G5NH_2_. Furthermore, the importance of Arg356^6.58^, Tyr189^4.60^, and His207^ECL2^ for CCK2R binding with gastrin was verified by the alanine scanning mutagenesis, which would completely abolish the binding of gastrin-17 (Zhang et al., 2021).

## 4 Conclusion

In this research, we performed extensive GaMD simulations to investigate the molecular determinants in the process of gastrin C-terminal pentapeptide amide end activating CCK2R. The C-terminal indispensable amide cap could significantly influence the binding modes of pentagastrin to CCK2R. Amidated pentagastrin has much similar binding modes as the counterpart in gastrin-17. Non-amidated pentagastrin moved its negatively charged C-terminus to interact with the positively charged Arg cluster (208^ECL2^, 213^5.33^, 215^5.35^, 356^6.58^, 365^ECL3^) distributed near the extracellular domain of CCK2R. Different binding modes induced different conformational dynamics of CCK2R. Residue pairs in CCK2R binding with amidated pentagastrin had stronger correlated motions than binding with non-amidated pentagastrin. 12 Key residues important for CCK2R-pentagastrin binding were identified. Among them, Arg356^6.58^, Arg215^5.35^, Tyr189^4.60^, and His207^ECL2^ formed strong salt bridges or hydrogen bonds with Asp in the amidated pentagastrin. Thus, they played dominant roles in constraining the position of Asp. In summary, our results explained the indispensable role of the C-terminal amide cap for gastrin’s bioactivity on a molecular basis. And the identified key interactions between the essential bioactive unit of gastrin and CCK2R would provide significant insights for developing CCK2R antagonists.

## Data Availability

The original contributions presented in the study are included in the article/Supplementary Material, further inquiries can be directed to the corresponding authors.
