# Supplementary material for: Elucidating the molecular determinants in the process of gastrin C-terminal pentapeptide amide end activating cholecystokinin 2 receptor by Gaussian accelerated molecular dynamics simulations
Source: Front Pharmacol. 2023 Jan 23;13:1054575. doi: 10.3389/fphar.2022.1054575 (PMC9899899; doi:10.3389/fphar.2022.1054575)
Supplement: Supplementary file 1 [file DataSheet1.doc]

**Elucidating the Molecular Determinants in the process of Gastrin C-terminal Pentapeptide Amide End Activating Cholecystokinin 2 Receptor by Gaussian Accelerated Molecular Dynamics Simulations**

**Kecheng Yang^1*^, Huiyuan Jin^2^, Xu Gao^1^**

^1^ National Supercomputing Center in Zhengzhou, Zhengzhou University, Zhengzhou, P. R. China

^2^ School of International Studies, Zhengzhou University, Zhengzhou, P. R. China

***Correspondence:**

yangkch@zzu.edu.cn


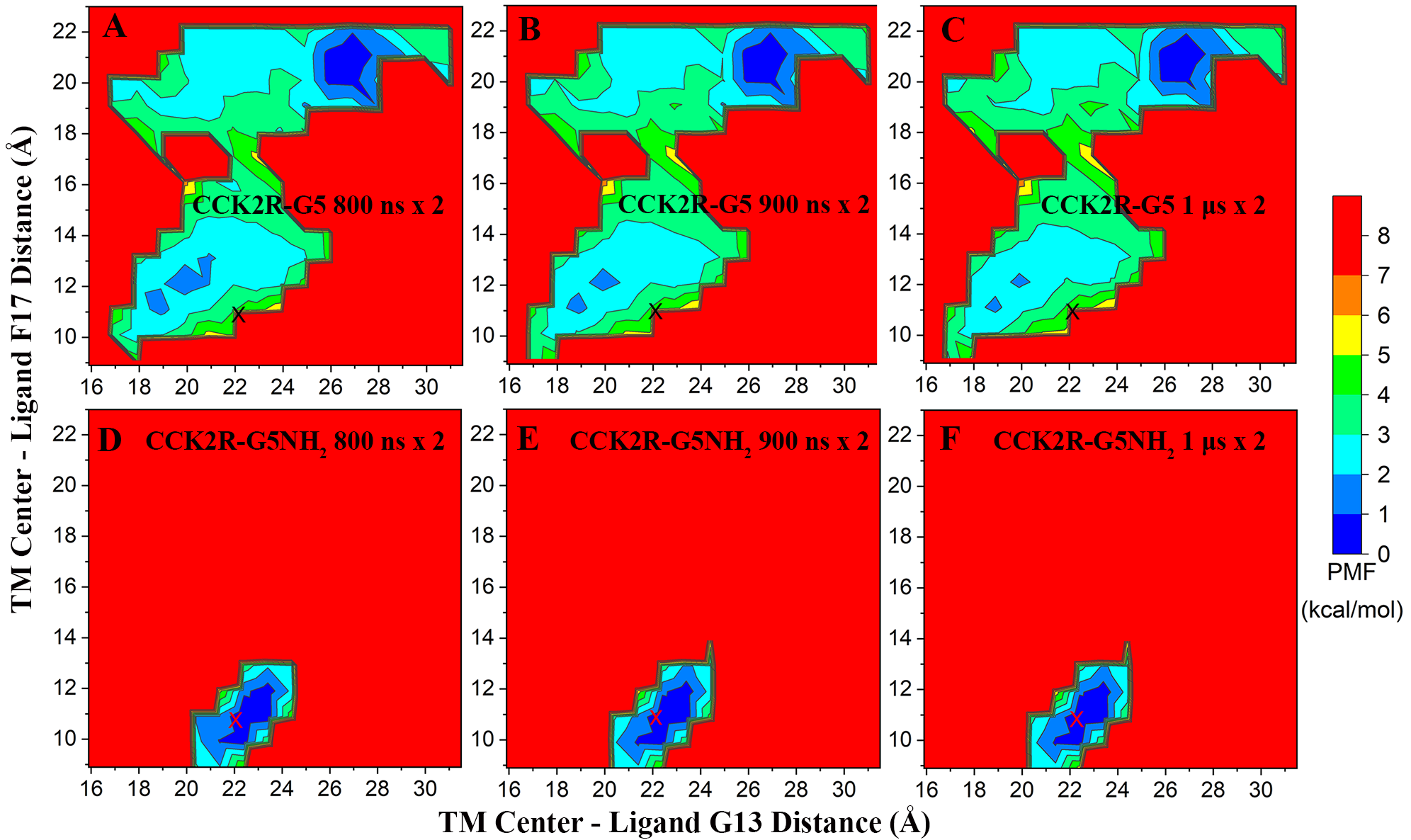


Figure S1. 2D PMF profiles of CCK2R-G5 (A-C) and CCK2R-G5NH_2_ (D-F) along the reaction coordinates of distances between the N/C-terminus of G5/G5NH_2_ (Cα atoms of G13 and F17, respectively) and the geometrical center of TM domain of CCK2R with two individual trajectory lengths of 800, 900, 1000 ns combined, respectively. The initial site from the cryo-EM structure was marked as symbol X (coordinate: 22.1, 10.8).


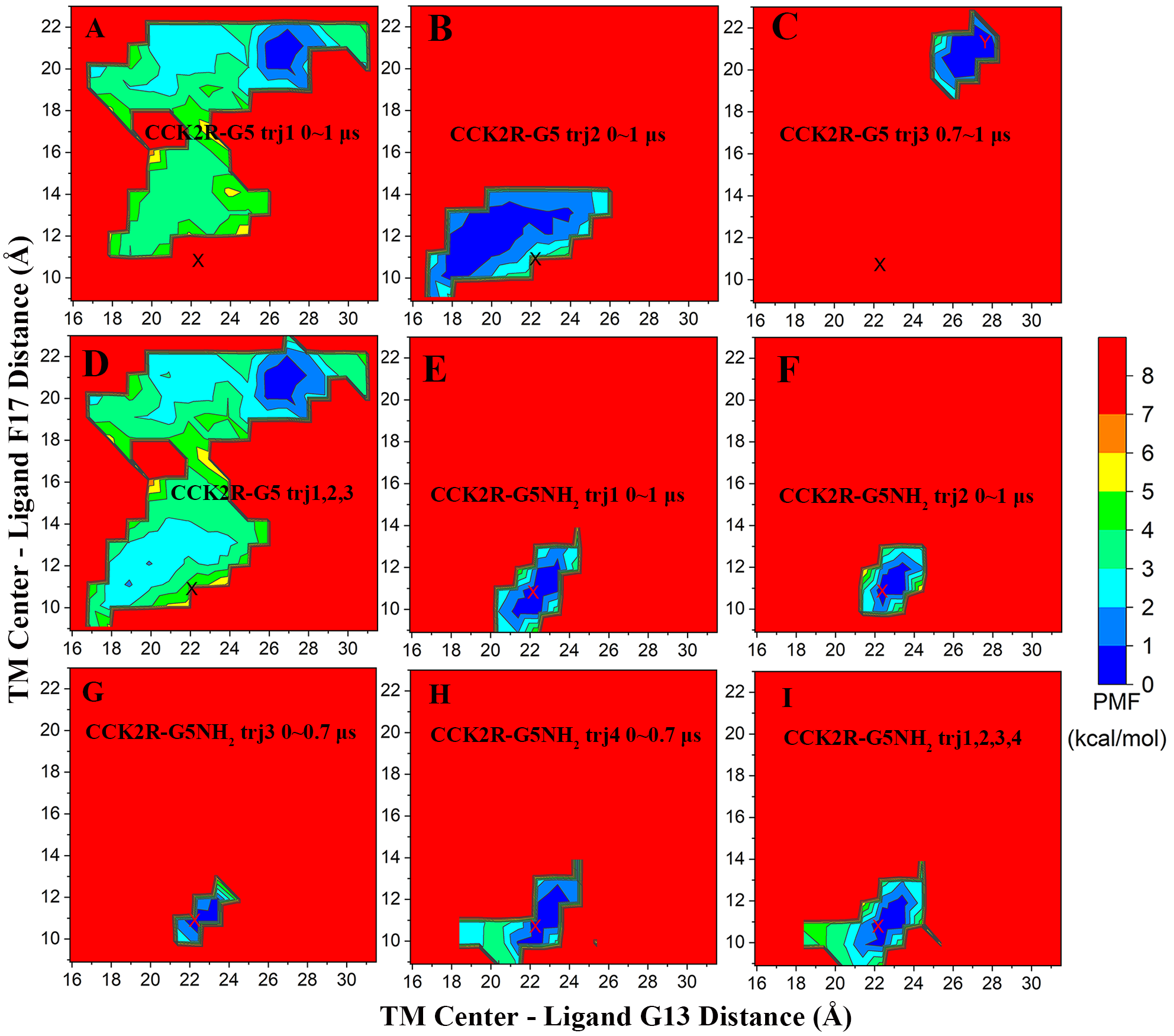


Figure S2. 2D PMF profiles of CCK2R-G5 (A-D) and CCK2R-G5NH_2_ (E-I) along the reaction coordinates of distances between the N/C-terminus of G5/G5NH_2_ (Cα atoms of G13 and F17, respectively) and the geometrical center of TM domain of CCK2R, respectively. Trajectory 3 for CCK2R-G5 (C) started from the conformational state (marked as symbol Y in C) at 700 ns of trajectory 1 with randomized initial atomic velocities; PMF profiles in D and I were calculated on the merged trajectories for each system. The initial site from the cryo-EM structure was marked as symbol X (coordinate: 22.1, 10.8).


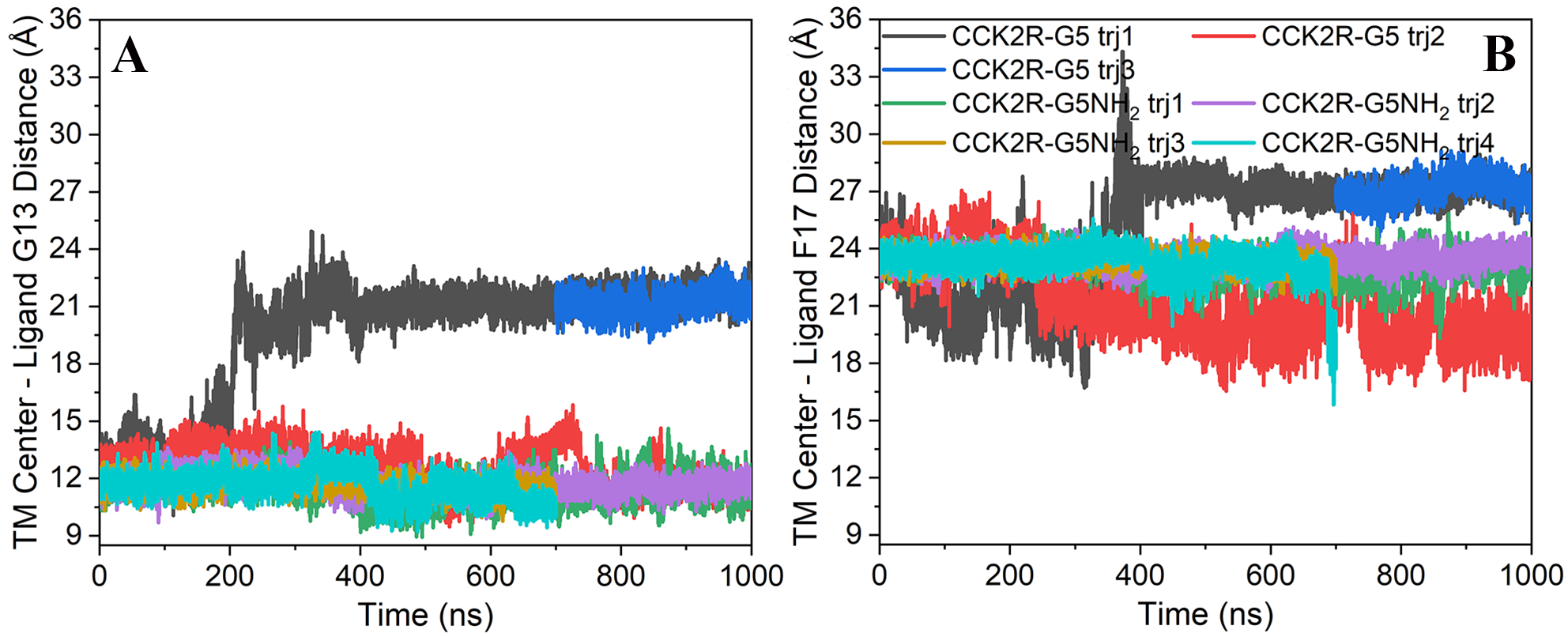


Figure S3. Time evolutions of two reaction coordinates in each simulation trajectory. All panels use the same legend listed in B.


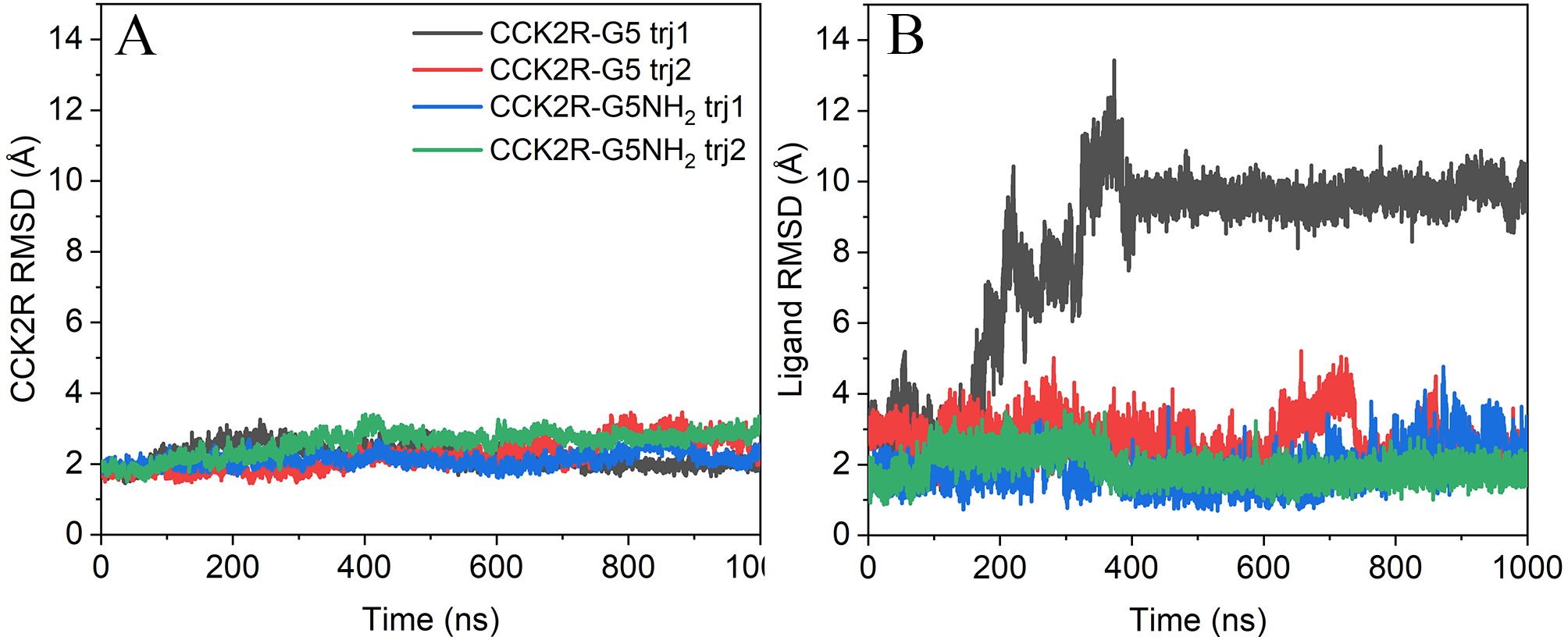


Figure S4. Time evolutions of RMSD values of simulation structures of CCK2R (A, based on TM backbone atoms) and G5/G5NH_2_ (B, based on backbone atoms) from their initial structures (aligned with the TM backbone atoms) in each simulation trajectory. All panels use the same legend listed in A.


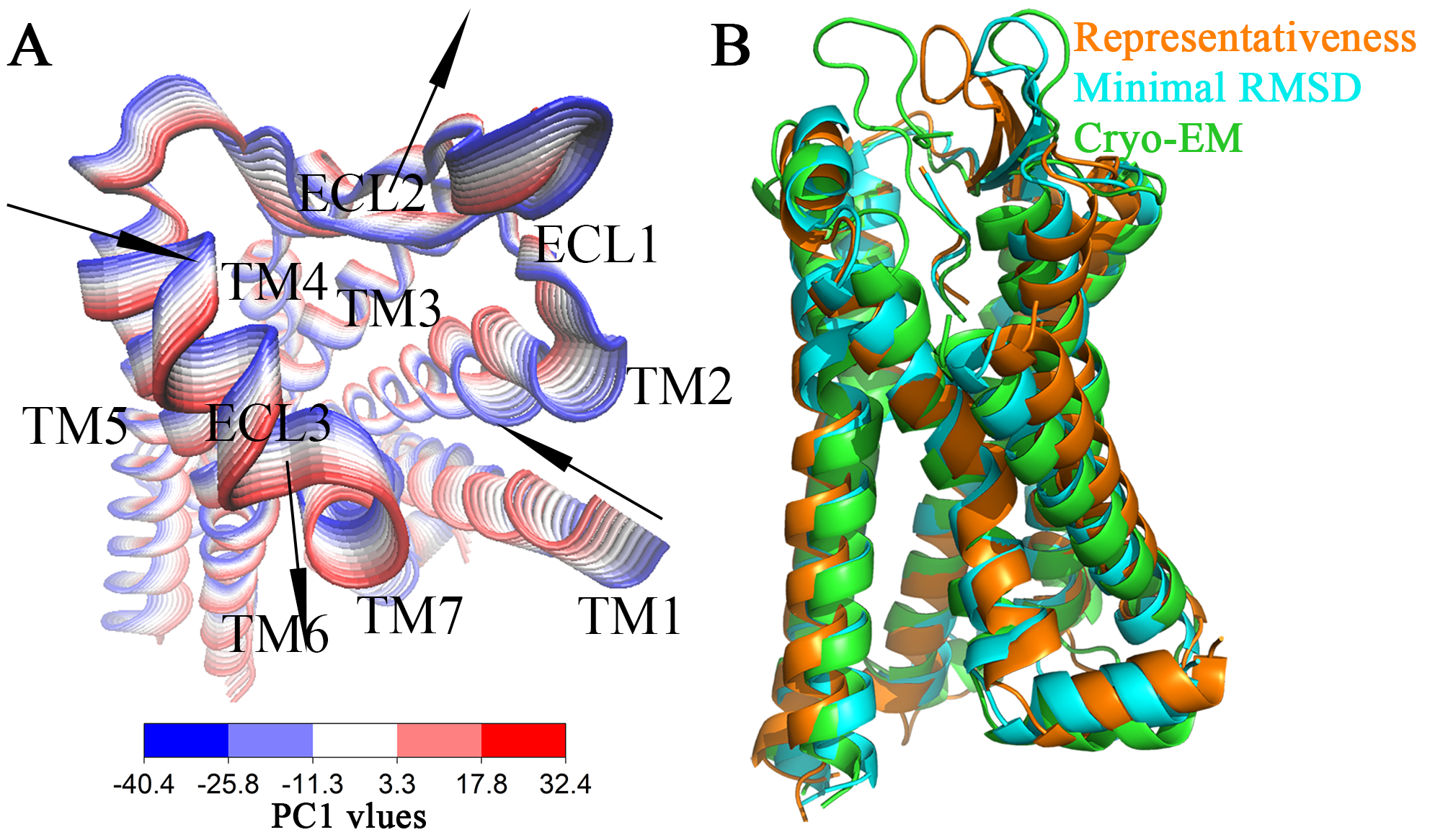


Figure S5. (A) Structural motions of CCK2R along with the PC1. Color scales represent a map of the PC1 values of structures. It should be noted that the structures with extreme colors were the projections of the corresponding CCK2R structures along the PC1, while other structures were generated to evenly fill the gap between these two projected structures. The purpose is to clearly show the structural motions from an extreme to the other. (B) Structural superimposition of two simulated CCK2R-G5NH_2_ models with minimal PMF values and the Cryo-EM structure of CCK2R-gastrin17 (green color, PDB code: 7F8W). One of the simulated structures is the representative structure used in Figure 2B (orange color), and the other is the structure with minimal RMSD of CCK2R from the Cryo-EM structure (cyan color, RMSD=1.51 Å).


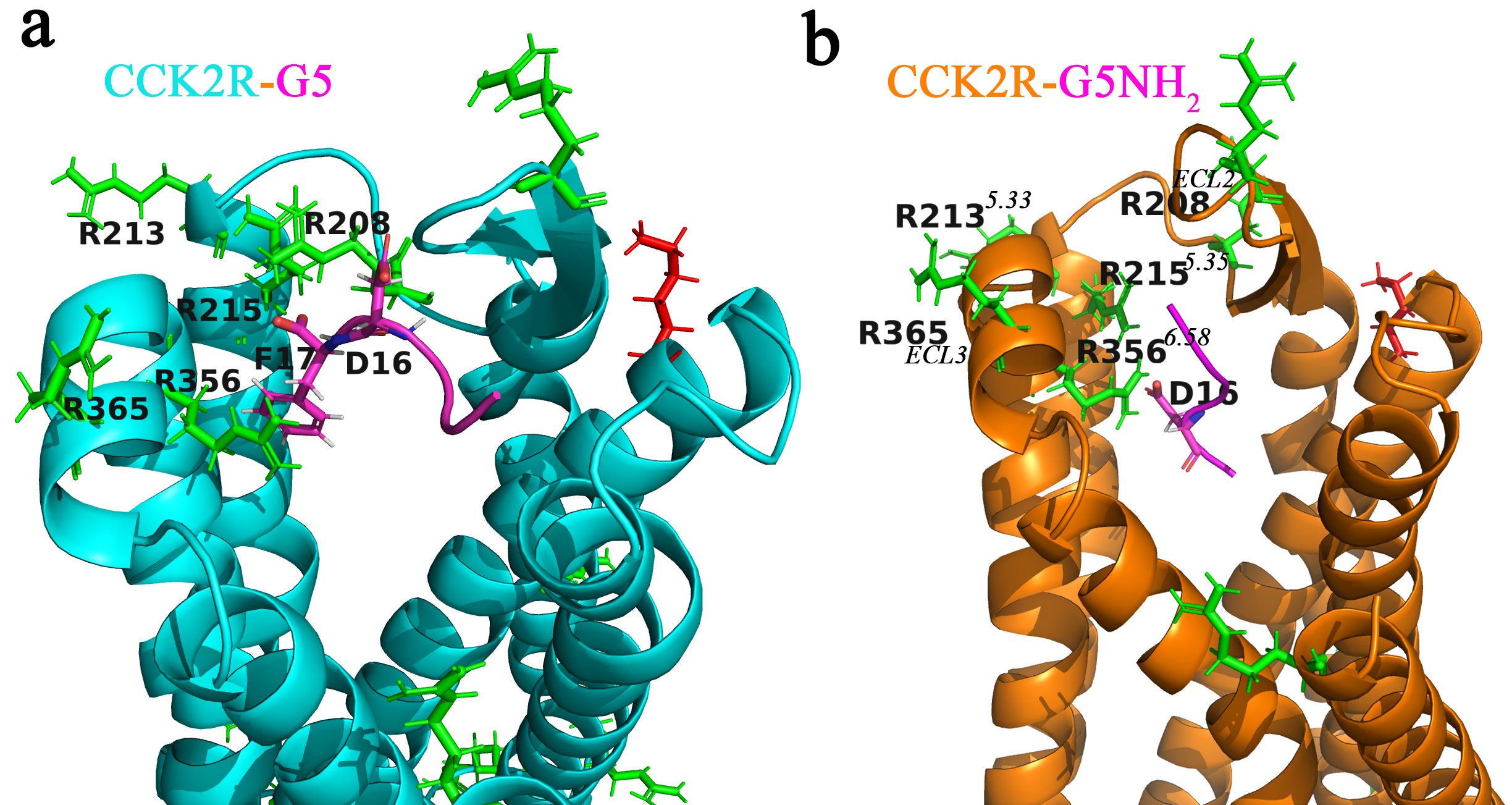


Figure S6. Charged residues in CCK2R-G5/G5NH_2_ complexes. Charged residues in CCK2R are shown in color stick representations and red for Arg, purple for Lys, respectively. Asp in G5NH_2_ and G5, and Phe in G5 are shown in sticks.


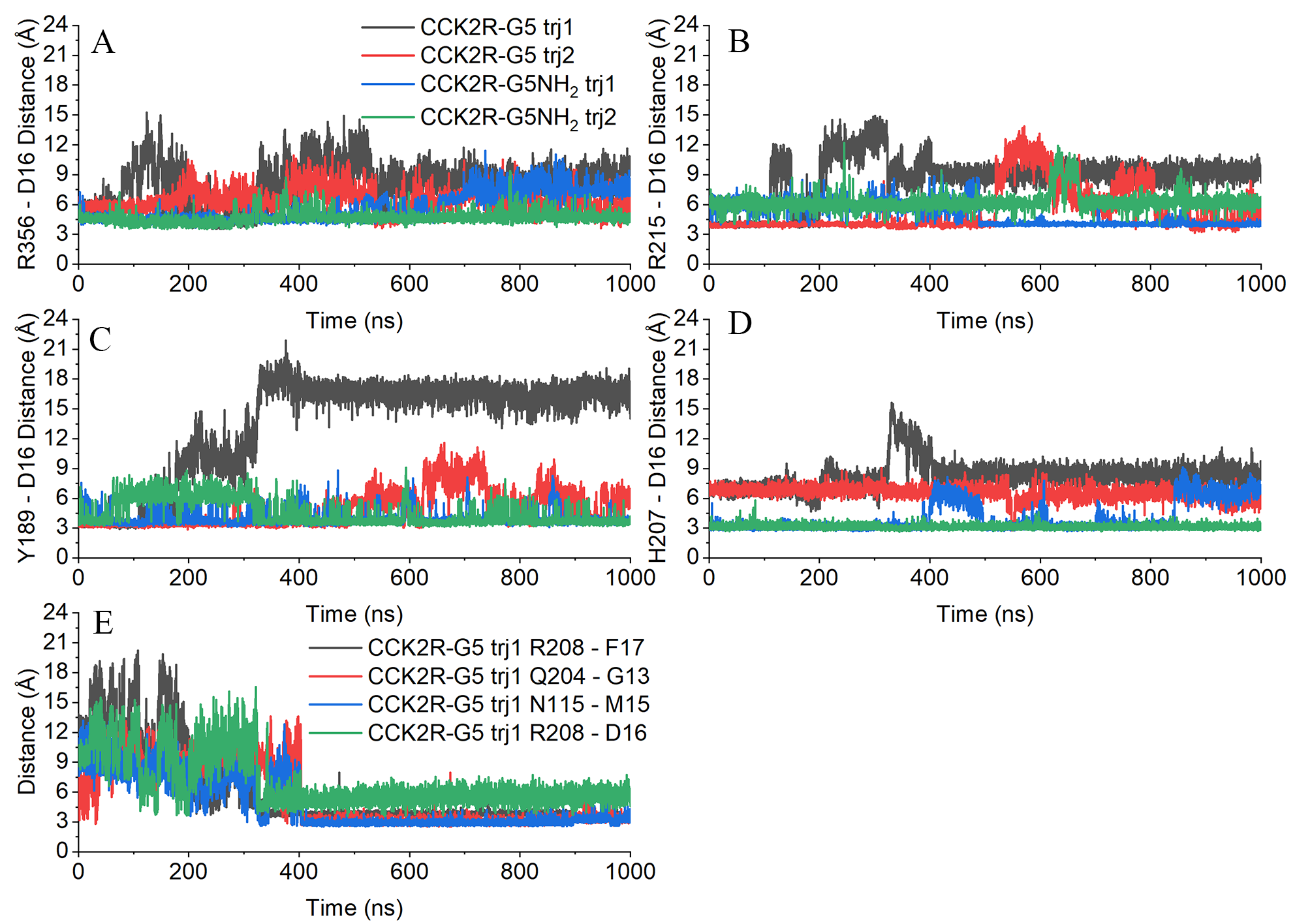


Figure S7. Time evolutions of distances between residue pairs with key interactions in the simulation structures. Panels A to D use the same legend listed in A. Distances between hydrogen bonds were calculated on the atoms of donor and acceptor (Figure 3E, F), and between salt bridge (i.e., Arg - Asp) was calculated on the C atom on guanidine group in Arg and C atom on carboxyl group in Asp.

Table S1. Definition of CCK2R transmembrane domains.

| Name | Region in the sequence |
| --- | --- |
| TM1 | Leu52-Ser82 |
| TM2 | Thr87-Gly118 |
| TM3 | Thr124-Arg158 |
| TM4 | Thr167-Thr193 |
| TM5 | Ser211-Leu247 |
| TM6 | Gln322-Asp359 |
| TM7 | Gly369-Met393 |

**Table S2.** MM-PBSA derived binding free energies (in unit of kcal/mol) of G5 and G5NH_2_ to CCK2R. MM-PBSA was calculated on the conformations of CCK2R-G5NH_2_ with minimal PMF value (same as Table 1) and on the conformations of CCK2R-G5 with similar binding modes as above CCK2R-G5NH_2_ conformations.

| **complex** | **ΔG_gas-vdw_** | **ΔG_gas-ele_** | **ΔG_sol-nonpolar_** | **ΔG_sol-polar_** | **ΔG_binding_** |
| --- | --- | --- | --- | --- | --- |
| CCK2R-G5 | -46.51(0.44) | -105.35 (0.32) | -6.44(0.01) | 100.00 (0.27) | -58.31(0.42) |
| CCK2R-G5NH_2_ | -58.35(0.10) | -61.95 (0.05) | -6.83(0.00) | 60.30(0.03) | -66.83(0.09) |

The standard error of the mean of the energy is shown in parentheses.

Table S3. Free energy (in unit of kcal/mol) contributions of residues in G5 (up) and G5NH_2_ (down) for binding with CCK2R. Values for the same residue with changes over 10 kcal/mol are shown in bold. ΔG_sol-nonpolar_ is not shown because this term is indecomposable in the current PBSA method.

| Residue | Δ*G_gas-vdw_* | Δ*G_gas-ele_* | Δ*G_sol-polar_* | Δ*G_binding_* |
| --- | --- | --- | --- | --- |
| Gly | -2.89(1.74) | -2.03(1.43) | 0.75(0.17) | -4.17(2.23) |
|  | -2.23(0.61) | -0.22(3.12) | 0.43(0.36) | -2.02(2.75) |
| Trp | -6.69(1.02) | -1.55(1.00) | 0.53(0.15) | -7.71(1.44) |
|  | -8.98(1.57) | -7.79(2.94) | 1.54(0.34) | -15.23(3.47) |
| Met | -4.71(2.51) | -4.34(1.59) | 0.64(0.20) | -8.41(2.82) |
|  | -7.20(0.80) | -2.33(1.32) | 0.42(0.15) | -9.10(1.39) |
| Asp | -3.59(0.57) | **-144.14(7.59)** | 23.41(1.19) | **-124.32(6.55)** |
|  | -1.74(1.75) | **-172.61(9.92)** | 26.91(1.06) | **-147.44(8.09)** |
| Phe | -2.18(8.87) | **-192.04(6.47)** | **29.99(0.88)** | **-164.22(7.55)** |
| Phe-NH_2_ | -9.04(0.78) | **-2.91(1.22)** | **1.07(0.21)** | **-10.88(-1.39)** |

The standard error of the energy is shown in parentheses.
